# A comprehensive safety evaluation of approved 2′-fucosyllactose for human nutritional applications in China

**DOI:** 10.3389/ftox.2026.1836564

**Published:** 2026-06-23

**Authors:** Jiangge Zheng, Huali Wang, Wenfeng Liu, Zhongzhou Chen, Jiyue Zhang, Jianbo Zhang

**Affiliations:** 1 Division III, China National Center for Food Safety Risk Assessment, Beijing, China; 2 State Key Laboratory of Agrobiotechnology, College of Biological Sciences, China Agricultural University, Beijing, China

**Keywords:** 2′-fucosyllactose, biosynthesis, exposure assessment, toxicological safety assessment, use levels

## Abstract

**Background:**

2′-Fucosyllactose (2′-FL), as a significant human milk oligosaccharide, plays a crucial role in infant formula products. This study aims to provide a scientific basis for the safe application of 2′-FL by systematically evaluating its physicochemical properties, production strain safety, toxicological data, and population exposure levels.

**Methods:**

This study characterizes the physicochemical properties of 2′-FL, evaluates the safety of its production strain, and conducts a series of toxicological assessments, including an acute oral toxicity test, genotoxicity tests, a 90-day subchronic toxicity test, and a teratogenicity test. Additionally, an exposure assessment of 2′-FL was conducted by integrating usage level data with food consumption data.

**Result:**

The production strain for 2′-FL is recognized as safe, and all the results of toxicological experiments show negative or no observed adverse effects. Exposure assessment data further confirm that infant and young child populations exposed to 2′-FL remain within established safety limits under the specified usage levels. Consequently, the use of 2′-FL as a food ingredient is considered safe.

**Discussion:**

The safety and technological necessity of 2′-FL constitute the scientific basis for its approval as a novel nutritional additive authorized for use in infant formula products.

## 1 Introduction

Human milk oligosaccharides (HMOs) are complex oligosaccharides with structural diversity in human milk, which is the third largest nutrient component in human milk after lactose and lipids (Newburg, 2013; Ballard and Morrow, 2013). They are typically composed of 3–14 monosaccharides and are decorated with a fucose or sialic acid residue attached to the lactose core via α1–2/3/4 and α2–3/6 linkages, respectively (Asakuma et al., 2011). HMOs possess prebiotics, immunomodulatory, and pathogen-antagonistic properties, and are closely related to the health benefits of breastfeeding in infants (Wiciński et al., 2020; Hill et al., 2021; Christian et al., 2021).

HMOs are synthesized in the mammary gland by the action of specific glycosyltransferases that sequentially add *N*-acetylglucosamine (GlcNAc), galactose (Gal), fucose (Fuc), and *N*-acetylneuraminic acid (Neu5Ac) to the basic acceptor molecule, lactose (Kobata, 1990). Human milk contains three major HMO types: fucosylated neutral HMOs (35%–50%); sialylated acidic HMOs (12%–14%), and non-fucosylated neutral HMOs (42%–55%) (Smilowitz et al., 2014), which are classified based on their terminal modifications. Both fucosylated and non-fucosylated HMOs fall under the category of neutral HMOs, while sialylated HMOs are considered acidic. Neutral HMOs account for more than 75% of the total HMOs (Plaz et al., 2018). 2′-FL is the most abundant neutral fucosylated oligosaccharide in human milk and has been extensively studied among HMOs (Vázquez et al., 2015). 2′-Fucosyllactose (2′-FL) is a synthetic trisaccharide consisting of L-fucose, D-galactose and D-glucose, which is produced by using L-fucose and D-lactose as starting raw materials. Many studies have shown that 2′-FL has numerous significant biological properties, including promoting the colonization and maintenance of symbiotic microbiota in the infant’s intestinal tract, establishing a balanced infants’ gut microbiota, strengthening the gastrointestinal barrier, preventing infections, and potentially supporting the immune system, brain, and cognitive development (Hill et al., 2021; Yu et al., 2013; Liu et al., 2024; Holscher et al., 2017; Wang Y. et al., 2020; Sprenger et al., 2022). Moreover, 2′-FL has been confirmed to enhance intestinal development and nutrient absorption (Nishikawa et al., 2026), and also exert inhibitory effects against age-related obesity and metabolic disorders by remodeling gut microbiota, repairing intestinal barrier function, and regulating the gut microbiome-T cell immune axis (Li et al., 2025).

Various advanced tools, such as synthetic biology, systems biology tools, and modular metabolic pathway assembly, have been explored to maximize the yield of target products (Faijes et al., 2019). The main ways to produce HMOs include chemical synthesis and microbial fermentation (Zhou et al., 2021; Petschacher and Nidetzky, 2016). The rapid advancements in synthetic biology and metabolic engineering have led to groundbreaking progress in the biosynthesis of HMOs via microbial cell factories. Currently, various genetically engineered strains are used as production strains to produce 2′-FL, such as *Escherichia coli* (Chin et al., 2015; Ni et al., 2020; Wang et al., 2023)*, Bacillus subtilis* (Zhang et al., 2025), *Saccharomyces cerevisiae* (Yu et al., 2018), *Pichia pastoris* (Fang et al., 2025), and *Corynebacterium glutamicum* (Lee et al., 2024), which have been developed for the efficient metabolic synthesis of HMOs. In addition to host selection, numerous studies have focused on optimizing microbial cell factories and engineering strategies for HMOs production, including transcriptional and translational regulation (Yu et al., 2022), elimination of redundant metabolic pathways (Lee et al., 2021), enhancement of product synthesis and transport (Zhu et al., 2021), as well as the optimization of fermentation strategies (Li et al., 2021). Collectively, these approaches contribute significantly to achieving targeted HMOs biosynthesis.

Since 2015, in the United States, the European Union, Australia, New Zealand and other countries or regions, some HMO products, including 2′-FL, 3-FL, LNnT, LNT, 3-SL, and 6-SL, have been approved by relevant authorities for use in some food categories, including infant formula. 2’-FL has been approved as “generally recognized as safe” (GRAS) by the Food and Drug Administration (FDA) and as a novel food by the European Food Safety Authority (EFSA). It is permitted to be added as a food ingredient to infant formula, and is also used in dietary supplements and medical foods. In China, it was approved as a nutritional additive by the National Health and Health Commission in China in 2023. This research elaborates on the characteristics of 2-FL from several aspects, including the production process, safety, and determination of usage amount. It also points out the differences in quality specifications and management ideas compared with those in other countries.

## 2 Results

### 2.1 Physical and chemical properties of 2′-FL

**FIGURE 1:**
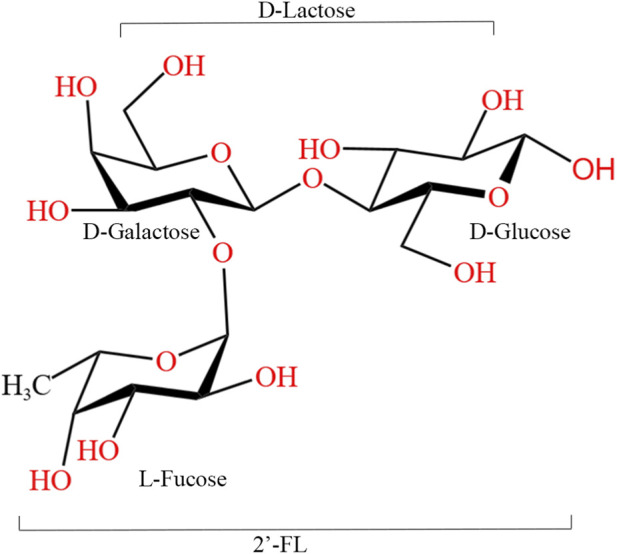
Molecular structure of 2′-FL.

2′-FL is a trisaccharide consisting of L-fucose linked via an α-(1–2′) bond to the D-galactose moiety of D-lactose (Figure 1). It is produced by genetically modified microorganisms through fermentation using lactose as the raw material, followed by multiple purification and drying processes. The chemical identity of 2′-FL is shown in Table 1. The molecular structure of 2′-FL was determined by liquid chromatography–tandem mass spectrometry (LC–MS/MS) based on the retention time, accurate mass and fragmentation pattern. The identity of 2′-FL produced by microbial fermentation was confirmed by high-performance anion-exchange chromatography (HPAEC), high-resolution mass spectrometry (HRMS), infrared (IR) and nuclear magnetic resonance (NMR) spectroscopy, showing that the 2′-FL produced by microbial fermentation has the same molecular weight, molecular formula and chemical structure as the 2′-FL found naturally in human milk.

**TABLE 1:** Chemical identity of 2′-FL.

Parameters	Indicators
Chemical name	α-L-Fucopyranosyl -(1→2)-β-D-galactopyranosyl -(1→4)-D-glucopyranose
CAS number	41,263–94-9
Molecular formula	C_18_H_32_O_15_
Molecular weight	488.44 Da
Color	white to almost white
Appearance	powder

### 2.2 Toxicological safety assessment of 2′-FL

To demonstrate the safety of 2**′**-FL, we conducted toxicological safety evaluation studies on 2′-FL, including an acute oral toxicity test, three studies on the potential mutagenicity of 2′-FL (a bacterial reverse mutation test, an *in vivo* mammalian erythrocyte micronucleus test, an *in vivo* mouse spermatogonia chromosomal aberration test), a 90-day subchronic toxicity test, and a teratogenicity test.

Throughout the 14-day observation period, no apparent toxic symptoms or mortality were observed in any animals. Body weight changes in both male and female animals are presented in Table 2. The results of the acute oral toxicity test showed that the oral tolerance dose and the median lethal dose (LD_50_) of 2′-FL were greater than 10.00 g/kgbody weight (g/kgbw) in female and male ICR mice. According to the acute toxicity grading evaluation standard, the test substance belongs to the practically non-toxic grade.

**TABLE 2:** The results of the acute oral toxicity test of 2’-FL on ICR mice (mean ± SD, *n* = 20).

Gender	Dose (g/kg·bw)	Animal weight (g)			Number of dead animals	LD50 (g/kg·bw)
		Initial	A week	Final		
Female	10	19.13 ± 1.06	23.06 ± 1.44	25.30 ± 1.65	0	>10
Male	10	20.57 ± 0.97	30.39 ± 1.57	34.92 ± 2.04	0	>10

The results of the bacterial reverse mutation test (Table 3) showed that the plate background bacterial moss grew well in each dose group, and the number of revertant colonies did not exceed twice the spontaneous control level. No mutagenic activity was observed. Thus, under the present experimental conditions, 2′-FL did not increase the revertant colony count in any of the five tested strains (TA97a, TA98, TA100, TA102, and TA1535).

**TABLE 3:** Bacterial reverse mutation test conducted with *S. involucrata* culture (mean ± SD).

​	Dose (μg/plate)	TA97a		TA98		TA100		TA102		TA1535	
​		-S9	+S9	-S9	+S9	-S9	+S9	-S9	+S9	-S9	+S9
2′-FL	8.0	116.0 ± 2.6	116.0 ± 2.6	31.7 ± 2.5	31.7 ± 5.9	156.7 ± 18.4	158.3 ± 7.6	255.7 ± 8.1	256.0 ± 10.8	22.7 ± 2.1	21.7 ± 1.2
	40.0	115.7 ± 1.2	116.0 ± 6.1	31.7 ± 0.6	31.7 ± 1.2	156.7 ± 12.7	157.3 ± 8.5	256.0 ± 16.8	257 ± 4.0	21.7 ± 1.2	21.7 ± 3.1
	200.0	114.7 ± 5.7	116.0 ± 10.5	31.7 ± 2.5	32.7 ± 2.5	157.7 ± 15.0	158.7 ± 9.6	256.0 ± 9.6	257.3 ± 15.9	21.3 ± 4.0	22.7 ± 1.2
	1,000.0	115.0 ± 10.0	115.0 ± 4.0	31.3 ± 3.2	32.0 ± 1.7	157.3 ± 4.7	157.7 ± 11.7	256.7 ± 8.6	257.0 ± 2.0	22.0 ± 3.6	22.0 ± 2.6
	5,000.0	115.0 ± 6.9	113.7 ± 4.2	32.0 ± 2.6	31.3 ± 5.9	158.7 ± 7.4	156.7 ± 13.9	256.3 ± 4.9	257.7 ± 6.1	21.3 ± 4.5	21.3 ± 0.6
Untreated control	—	117.0 ± 2.0	116.3 ± 8.3	32.0 ± 1.0	31.7 ± 1.5	157.0 ± 5.3	159.0 ± 14.1	255.7 ± 16.2	257.3 ± 14.6	22.3 ± 1.2	22.3 ± 1.5
H_2_O	—	115.3 ± 2.5	115.7 ± 2.5	31.7 ± 1.2	31.7 ± 4.9	157.3 ± 4.0	158.3 ± 4.2	255.7 ± 4.2	255.7 ± 3.1	21.7 ± 1.2	22.3 ± 2.5
Solvent control (DMSO)	—	116.0 ± 1.7	116.0 ± 3.6	31.7 ± 2.9	31.3 ± 0.6	158.7 ± 12.3	158.7 ± 13.4	255.3 ± 6.5	255.7 ± 11.4	22.0 ± 3.0	22.3 ± 1.2
2- Amino fluorene	10.0	​	1900.7 ± 145.7	​	1,411.3 ± 139.7	​	1,115.3 ± 89.0	​	​	​	​
Natriumazid	1.5	​	​	​	​	1,128.0 ± 121.0	​	​	​	585.3 ± 49.2	​
Dexon	50	2,582.7 ± 22.5	​	1,121.3 ± 112.4	​	​	​	1,130.7 ± 122.7	​	​	​
1,8-Dihydroxyanthraquinone	50	​	​	​	​	​	​	​	1,065.3 ± 122.7	​	​
Cytoxan	20	​	​	​	​	​	​	​	​	​	1,024.7 ± 38.1

Abbreviations: S9 = without metabolic activation; +S9 = with metabolic activation.

The mammalian erythrocyte micronucleus test results are shown in Supplementary Figure S1. Compared with the negative control group, the micronucleus cell rate of both female and male mice in the positive control group (cyclophosphamide) showed a statistically significant difference (*P* < 0.01). No significant differences were observed in the micronucleus cell rate in any dose groups of 2′-FL when compared with the negative control group (*P* > 0.05). These results indicate that 2′-FL did not induce an increase in the rate of micronucleated polychromatic erythrocytes in mouse bone marrow cells.

The mouse spermatogonia chromosome aberration test results are shown in Supplementary Figure S2. Compared with the negative control group, the chromosome aberration in the positive control group (cyclophosphamide) showed a statistically significant difference (*P* < 0.01). No significant differences were observed in the micronucleus cell rate in any dose groups of 2′-FL when compared with the negative control group (*P* > 0.05). There was no dose-response relationship between different doses, suggesting that the 2′-FL had no teratogenic effect on the chromosomes of mouse spermatogonia.

**FIGURE 2:**
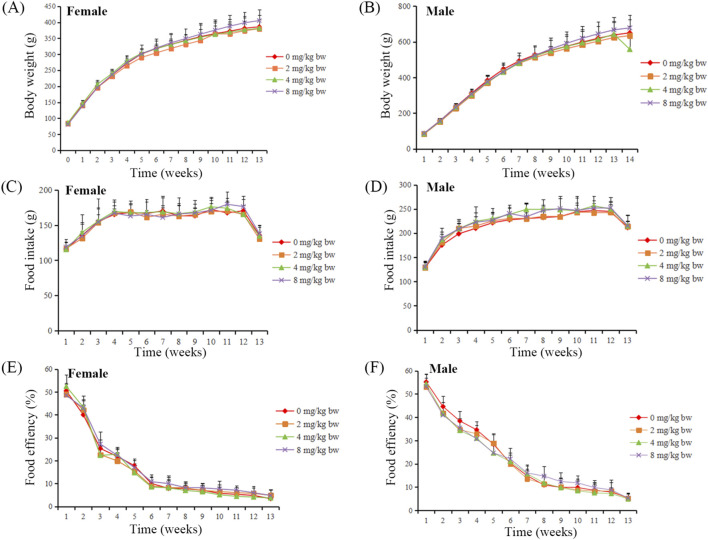
Weekly mean body weight of females **(A)** and male rats **(B)**, weekly mean daily food intake of females **(C)** and male rats **(D)**, weekly mean food efficiency of females **(E)** and male rats **(F)** in the 90-day feeding study. There were no significant differences in body weight, weekly food intake, or food efficiency in each dose group both the female and male rats compared with the negative control group (*P* > 0.05).

**FIGURE 3:**
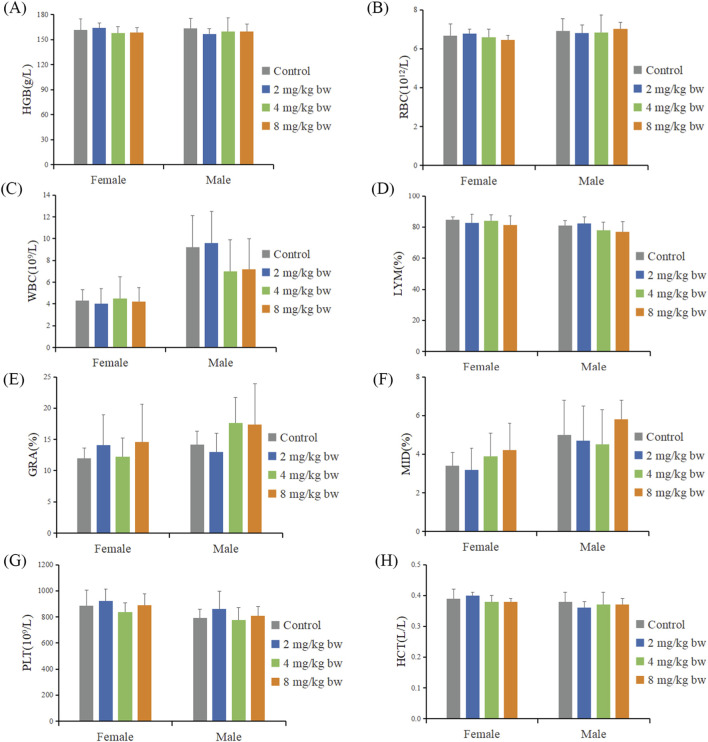
Hematological changes in male and female rats after 90 days. Hematological indices were assessed in rats, including **(A)** Hemoglobin (HGB), **(B)** Red blood cell Count (RBC), **(C)** White blood cell count (WBC), **(D)** Lymphocyte count (LYM), **(E)** Granulocyte count (GRA), **(F)** Mid-sized cells (MID), **(G)** Platelet count (PLT), and **(H)** Hematocrit (HCT). There were no significant differences in the hematological parameters in each dose group both the female and male rats compared with the negative control group (*P* > 0.05).

During the entire 90-day subchronic toxicity study, the growth of female and male rats in each measurement group remained healthy. No obvious abnormalities or behavioral changes were detected. As shown in Figure 2, there were no significant differences (*P* > 0.05) in body weight, food intake or food efficiency in each week relative to negative controls. Serum biochemical and hematological parameters were shown in Figure 3 and Table 4. We found no significant differences (*P* > 0.05) in these parameters over the course of the 90-day subchronic toxicity study. Absolute and relative main organ weights are shown in Supplementary Table S1. No significant differences (*P* > 0.05) in these parameters were detected between the control and high-dose groups. Based on the observations from a subchronic 90-day toxicity study in rats, the study proposed the high dose level of 8,000 mg/kg body weight as the no observed adverse effect level (NOAEL). In the teratogenicity study, oral administration of 2′-FL at doses of 2.0, 4.0, and 8.0 g/kg body weight to pregnant rats from gestational days 6–20 did not induce any observable maternal toxicity or embryotoxicity (data not shown).

**TABLE 4:** Serum clinical chemistry of rats on the 90th day in the subchronic test (mean ± SD).

Serum biochemistry	Control	Low	Middle	High
Female				
ALT (U/L)	37.6 ± 10.80	34.6 ± 9.30	33.2 ± 4.70	29.2 ± 6.80
AST (U/L)	137.3 ± 26.70	112.8 ± 30.20	125.4 ± 30.8	122.0 ± 22.40
TP (g/L)	74.2 ± 6.30	73.5 ± 4.00	72.3 ± 4.00	70.4 ± 3.60
ALB (g/L)	31.9 ± 3.40	31.2 ± 2.80	31.1 ± 2.40	29.8 ± 2.10
TC (mmol/L)	1.81 ± 0.44	1.62 ± 0.30	1.53 ± 0.23	1.55 ± 0.35
TG (mmol/L)	0.47 ± 0.15	0.38 ± 0.10	0.43 ± 0.15	0.38 ± 0.07
Glu (mmol/L)	5.73 ± 1.32	5.11 ± 1.12	5.06 ± 1.58	5.68 ± 0.98
BUN (mmol/L)	5.83 ± 0.67	5.85 ± 0.69	5.67 ± 1.09	5.83 ± 0.57
CR (μmol/L)	63.9 ± 3.60	60.8 ± 4.10	59.2 ± 6.30	59.9 ± 4.80
GGT (U/L)	6.4 ± 0.40	6.1 ± 0.40	6.4 ± 0.90	6.2 ± 1.00
ALP (U/L)	56.4 ± 15.20	55.4 ± 14.60	54.6 ± 15.60	62.4 ± 18.10
K (mmol/L)	4.84 ± 0.35	5.00 ± 0.31	4.89 ± 0.33	4.63 ± 0.28
Na (mmol/L)	152.6 ± 1.90	151.9 ± 3.00	152.4 ± 3.60	151 ± 8 3.10
Cl (mmol/L)	112.9 ± 2.10	114.2 ± 2.70	114.6 ± 3.90	112 ± 8 2.40
Male				
ALT (U/L)	42.9 ± 6.00	46.3 ± 4.50	42.2 ± 6.70	39.3 ± 7.80
AST (U/L)	153.3 ± 33.90	138.0 ± 23.60	133.5 ± 23.70	124.7 ± 32.90
TP (g/L)	64.9 ± 2.40	65.6 ± 2.80	65.8 ± 2.60	66.4 ± 3.20
ALB (g/L)	25.8 ± 1.20	26.5 ± 1.30	26.5 ± 1.10	26.5 ± 1.20
TC (mmol/L)	1.32 ± 0.41	1.36 ± 0.24	1.26 ± 0.40	1.26 ± 0.29
TG (mmol/L)	0.51 ± 0.25	0.51 ± 0.25	0.54 ± 0.28	0.40 ± 0.16
Glu (mmol/L)	8.03 ± 1.24	8.75 ± 1.58	7.64 ± 1.55	7.50 ± 1.46
BUN (mmol/L)	5.15 ± 1.51	6.09 ± 0.94	4.72 ± 0.72	5.45 ± 0.81
CR (μmol/L)	56.7 ± 2.40	59.8 ± 4.50	53.8 ± 3.60	57.8 ± 3.40
GGT (U/L)	8.1 ± 3.40	6.7 ± 1.00	6.3 ± 0.70	7.0 ± 1.10
ALP (U/L)	99.9 ± 22.10	102.5 ± 9.60	91.7 ± 19.60	93.6 ± 18.8
K (mmol/L)	5.00 ± 0.33	5.02 ± 0.35	4.80 ± 0.27	5.05 ± 0.15
Na (mmol/L)	143.2 ± 1.30	142.0 ± 1.40	142.8 ± 1.10	142.7 ± 0.90
Cl (mmol/L)	102.5 ± 1.90	102.7 ± 1.20	103.6 ± 1.00	103.3 ± 0.90

### 2.3 Safety assessment of 2′-FL production strain and final product

It has been demonstrated that microbial production of 2′-FL is feasible to scale up using engineered strains. Many studies developed synthetic biology approaches to promote the efficient biosynthesis in engineered *E. coli*, which is the most common host for the production of GDP-fucose for 2′-FL production (Chin et al., 2017; Huang et al., 2017; Jung et al., 2019). The production strain used in this study was *E. coli* BL21 (DE3). The final 2′-FL product was subjected to multiple purification steps, including ultrafiltration, microfiltration, ion exchange, and sterile filtration. PCR analysis showed no detectable exogenous gene DNA fragments in the final product (Supplementary Figure S3). Plate streaking further demonstrated that no viable genetically modified production strains were present in the final product (Supplementary Figure S4).

In addition to demonstrating the absence of exogenous gene residues and viable bacterial residues in the final 2′-FL product, the safety of the production strain, the recipient microorganism, and the genetic manipulation procedures was also confirmed in accordance with the ‘Requirements for Application Dossiers for Safety Evaluation of Genetically Modified Microorganisms Used in Food Processing' issued by the National Health Commission (data not shown). The relevant experimental results indicate that the genetically edited microorganisms show no safety differences compared with the non-edited recipient microorganisms.

### 2.4 The use levels of 2′-FL in approved food categories

The content and composition of HMO in breast milk are affected by lactation stages, secretor status of mothers and dietary patterns (Elwakiel et al., 2018). Studies show that the HMO concentrations are highest in colostrum, followed by slightly lower concentrations in transitional milk, with a gradual decline in mature milk as lactation progresses (Thum et al., 2021). Significant differences in HMO composition have been described between countries. In China, 70%–80% of breast milk is secretory breast milk (Elwakiel et al., 2018; Thum et al., 2021; Bai et al., 2018; Wang M. et al., 2020). 2′-FL is the most abundant oligosaccharide in human milk, accounting for about 30% in HMO, and the content of 2′-FL varies greatly in different breast milk types.

The proposed maximum intake level of the 2′-FL by infants is within the range of 2′-FL levels found in mature breast milk, as reported in the literature. This study collected relevant reports on the analysis of breast milk samples in China, including Beijing, Shanghai, Suzhou, Guangzhou, Xuchang and other regions. Through the relevant research on the levels and distribution of HMOs in Chinese breast milk, this research provided a scientific basis for formulating appropriate intake levels of breast milk oligosaccharides that meet the needs of Chinese infants and young children.

In this study, the maximum intake level of the 2′-FL for infants was set within the range of 2′-FL levels found in mature breast milk in China. The natural concentration of 2′-FL in human milk in China is shown in Table 5. The concentration range of 2′-FL in human milk is relatively wide, with the maximum reaching 2.93 g/L (Zhou et al., 2021), 3.02 g/L (Wu et al., 2020), and 2.89 g/L (Liu et al., 2021). Based on the natural concentration of human milk samples in China, the mean concentration, i.e., 2.4 g/L, is set as the maximum amount of use that is nutritionally effective for infants, which is still under the highest mean concentrations observed naturally in Chinese breastmilk, which may reach as high as 3 g/L (Zhou et al., 2021; Wu et al., 2020).

**TABLE 5:** 2′-FL concentrations (g/L) in human colostrum, transitional, and mature milk from mothers with either positive or unknown secretor status (mean ± SD).

References	Colostrum	Transition	Mature				
Zhou et al. (2021)	2.93 ± 1.08 (1–7 days)	1.93 ± 0.57 (8–14 days)	1.72 ± 0.45 (15–60 days)	1.19 ± 0.26 (61–120 days)	0.93 ± 0.19 (>121 days)	​	
Wenkui Guo et al. (2021)	0.97 ± 0.09 (1–12 days)	nr	0.74 ± 0.19 (13–30 days)	nr	0.69 ± 0.09 (>60 days)	​	
Yuan’an Wei et al. (2017)	2.27 (3 days)	nr	nr	nr	nr	0.22 (202 days)	
Austin et al. (2016)	nr	2.6 ± 0.97 (5–11 days)	2.3 ± 0.8 (12–30 days)	2.2 ± 0.73 (30–60 days)	1.8 ± 0.67 (60–120 days)	1.3 ± 0.51 (120–240 days)	
Zhu et al. (2017)	2.9 ± 1.38 (0–180 days)						
Ma et al. (2018)	nr	1.28 ± 1.05 (14 days)	1.37 ± 1.12 (30 days)	1.18 ± 1.02 (60 days)	0.98 ± 0.89 (90 days)	0.70 ± 0.75 (180 days)	0.71 ± 0.67 (240 days)
Huang et al. (2019)	1.70 ± 1.10	1.51 ± 0.90	1.399 ± 0.86				
Wu et al. (2020)	3.02 (3 days)	2.54 (5–10 days)	2.35 (11–30 days)	1.96 (31–60 days)	1.56 (61–100 days)	1.28 (168 days)	
Liu et al. (2021)	2.89 (0–5 days)	2.16 (10–15 days)	2.06 (40–45 days)	1.03 (200–240 days)	1.01 (300–400 days)	​	

Furthermore, 2′-FL should be added in sufficient amounts in food to achieve the intended effect, i.e., selecting a value that will bring infant formula closer to a representative composition of Chinese breastmilk. The minimum intake level of the 2′-FL was set at 0.7 g/L, which is the minimum for the mean 2′-FL content in human milk (mature milk) in China. Studies have shown that the average minimum value of 2′-FL in Chinese breast milk during 1–2 months of lactation is 0.69 g/L (Austin et al., 2016). The average content of 2′-FL in Chinese breast milk samples at 240 days postpartum is 0.71 g/L, and the content at 180 days postpartum is 0.70 g/L (Ma et al., 2018). Moreover, studies have shown that 2′-FL content decreased gradually with the prolongation of the lactation period; the average content in colostrum (1–12 days) was 0.97 ± 0.09 g/L, and the average content in transition milk (13–30 days) was 0.74 ± 0.19 g/L. The average content in mature milk (after 60 days) was 0.69 ± 0.09 g/L (Wenkui Guo and Liu, 2021). It can be seen that the minimum mean 2′-FL content in different lactation periods is about 0.7 g/L. Therefore, the minimum value is set at 0.7 g/L, establishing the minimum threshold of 2′-FL as a valuable functional ingredient, and ensuring that products containing 2′-FL can theoretically deliver the intended health benefits.

Based on these studies, the use levels for the 2′-FL as a nutritional additive in approved food categories are determined to be 0.7 g/L∼2.4 g/L, calculated by pure product, by ready-to-eat state. If 2′-FL is added to powdered products, the usage amount shall be converted based on the reconstitution ratio. Considering that galactooligosaccharides, fructooligosaccharides, polyfructose, raffinose, and the simultaneously approved (LNT) are all dietary fiber substances permitted in infant formula powder, a total content control is implemented for these substances to avoid potential gastrointestinal discomfort symptoms that may arise from their cumulative use. The total amount shall not exceed 64.5 g per kilogram.

### 2.5 Exposure assessment analysis of 2′-FL

To verify the safety of 2-FL, we further conducted a theoretical exposure assessment on 2′-FL, which was calculated based on its maximum usage in the food category approved and individual consumption data. The food consumption data was derived from the 2018 China Residents’ Food Consumption Survey. The theoretical exposure per kilogram of body weight per person per day was calculated using a simple distributed assessment model, combined with data on the range and limit of use in foods. The mean, highest, 95th and 99th percentile estimated daily intakes of the 2′-FL for infants aged from 0 to 3 years are presented in Table 6. The theoretical exposure assessment results showed that the general infant population of China had the mean and P95th percentile all-user intakes at 9.81 mg/kgbw and 26.40 mg/kgbw, respectively. 2′-FL intakes from infant formula could lead to an intake of about 34.44 mg/kgbw at the 95th percentile and 17.464 mg/kgbw at the mean for infants aged from 0 to 3 years.

**TABLE 6:** Results of the estimated per kilogram body weight intake of 2′-FL from proposed food categories by the infant population in China.

Sex/Age	All-person consumption (mg/kg·bw)			All-user consumption (mg/kg·bw)		
	Mean	P95	P99	Mean	P95	P99
0∼3 years (boy)	9.64	26.088	61.99	17.23	34.25	73.58
0∼3 years (girl)	9.99	26.67	68.74	17.73	34.61	82.29
Total	9.81	26.40	64.25	17.46	34.44	78.28

The exposure boundary (MOE) method was used to assess the exposure risk in this study. According to the NOAEL of 8,000 mg/kgbw obtained in the 90-day subchronic toxicity study, the MOE value of the average exposure and the P95th exposure of the consumer population could be calculated to be 441 and 224, respectively. The MOE value is greater than 100, indicating that the safety risk of 2′-FL is relatively low and within an acceptable range.

## 3 Discussion

2′-FL produced either through chemical synthesis or via fermentation using genetically engineered strains, has already been authorized as a novel food in the EU (Commission Implementing Regulation 2017/2470). In the United States, 2′-FL is recognized as “Generally Recognized as Safe” (GRAS) under its intended conditions of use (GRN NO. 650). Food Standards Australia New Zealand (FSANZ) has assessed an application made by Glycom A/S to permit the voluntary addition of 2′-FL alone or in combination with Lacto-N-neotetraose (LNnT), produced by microbial fermentation, in infant formula products and formulated supplementary foods for young children (Application A1155). The regulatory approaches for 2′-FL approval vary across countries. The following mainly analyzes the differences in terms of use levels, application scopes, and quality specifications.

The regulatory framework for 2′-FL in China differs significantly from that of other countries, being both more comprehensive in scope (setting minimum and maximum limits) and directly supported by the toxicological data from the present study. There is only a maximum limit requirement for the approval of 2′-FL in other countries, and the maximum levels vary across countries, 1.2 g/L (such as European Union, Singapore, and Canada) and 2.4 g/L (such as the United States, Australia/New Zealand) (Safety of 2′‐O‐fucosyllactose as a novel f ood ingredient pursuant to Regulation EC No 258/97, 2015; Bych et al., 2019). In contrast, China’s regulatory framework for 2′-FL application stipulates both maximum and minimum permissible limits, thereby establishing a defined range for safe and effective utilization. The setting of the maximum usage amount ensures product safety, while setting the minimum usage amount guarantees functional efficacy, safeguarding consumer rights and maintaining equitable market practices. The exposure assessment conducted in the present study (Section 2.5) demonstrated that the estimated daily intake of 2′-FL at the proposed maximum use level (2.4 g/L) remained well below the established NOAEL from the 90-day subchronic toxicity study, confirming that the Chinese regulatory limits are conservatively set within a safe margin. Furthermore, the current approval of 2′-FL in China is restricted exclusively to infant and young child food categories, which differs from other countries such as the United States, European Union, and Australia/New Zealand, where 2′-FL is permitted in both infant formula and some general food products. In the future, 2′-FL is expected to be applied to a broader range of food categories.

The quality specifications for 2′-FL vary across different regulatory jurisdictions, with China adopting a unique unified national standard in contrast to the multiple standards observed in other countries. The United States, European Union, Australia, and New Zealand have established quality specifications for 2′-FL produced via fermentation. These specifications not only set limits on the content of 2′-FL but also control impurities such as D-lactose (≤3.0% *w/w* DM) and difucosyllactose (≤2.0% *w/w* DM). Additionally, the quality standards impose restrictions on water content, ash content, heavy metals (lead, total arsenic), pathogenic microorganisms (Total plate count, Enterobacteriaceae, *Salmonella*), as well as residual endotoxins and residual proteins to ensure product safety. The GRAS declaration in the United States is issued by individual companies, functioning primarily as an internal corporate standard. Consequently, the quality specifications vary. In the EU, quality specifications for 2′-FL products stipulate varying parameters depending on the notifying entity, with content requirements ranging from no less than 94.0% to no less than 83.0%. In Australia and New Zealand, the primary content requirement for 2′-FL products is set at 94.0% or higher. In contrast, according to China’s management system for new food additives, each food additive typically adheres to a single quality specification, that is, national standard, which serves as the fundamental requirement for product quality and safety. Consequently, distinct from regulatory approaches adopted by other nations, 2′-FL in China is subject to a single, unified quality specification, which is set forth in Announcement No.8 (2023) of the National Health Commission (NHC). All 2′-FL products submitted by various manufacturers must comply with this unified quality requirement, and the 2′-FL products used in this study also meet this unified quality requirement, including the production process, physicochemical indicators, and analytical testing methods.

Regarding 2′-FL produced through fermentation using various genetically modified microbial strains, different countries adopt diverse approaches to announce the production strains. In the United States, the FDA’s official website announcement lists the production strain numbers, including those named internally by the company. In the EU regulation, the production strains allowed for the production of 2′-FL are listed only to the substrain level (such as K-12 or BL21), with explicit designation as genetically modified microorganisms. Australia/New Zealand regulations similarly specify strains at the subspecies level (such as K-12) while additionally listing gene donor organisms for representative enzymes. In China, food additive approvals are not specific to a particular manufacturer. Once authorized, all manufacturers must comply with the regulations. Consequently, all commercially produced 2′-FL must meet the published unified quality specification. Only the microbial information for different production strains used by different manufacture is continuously updated in the appendix to the announcement, including the production strains and the gene donor organisms. The initial step in HMOs biosynthesis involves the accumulation of intracellular sugar donors (such as GDP-fucose), followed by the second step wherein glycosyltransferases accurately recognize both glycosyl donors and acceptors to facilitate glycosyl transfer and subsequent assembly. Although the biosynthesis of 2′-FL involves multiple enzymes, only the names of microbial strains expressing the core enzyme (α-1,2-fucosyltransferase) are listed in the production strain documents.

The absence of body weight changes in both male and female animals following 2′-FL administration in the toxicological study is a noteworthy finding, particularly when considered in light of recently reported functional effects of 2′-FL. Previous studies have demonstrated that 2′-FL enhances intestinal development and nutrient absorption, while others have reported that 2′-FL may suppress age-related obesity. Enhanced nutrient absorption could theoretically raise concerns about undesirable weight gain. However, our finding that even high-dose 2′-FL administration did not induce body weight increases supports the concept that 2′-FL may enhance nutrient absorption while maintaining metabolic health, without promoting excessive weight gain. The observation that enhanced absorption does not lead to weight gain may be explained by the possibility that 2′-FL promotes nutrient absorption without disrupting energy balance, that the absorbed nutrients may exert metabolically regulatory effects rather than simply contributing to caloric surplus, or that 2′-FL modulates gut microbiota in a manner that favors metabolic health. Future studies should further explore the underlying mechanisms of the metabolic effects of 2′-FL, including its long-term impact on energy homeostasis across different population groups.

## 4 Experimental methods

### 4.1 Production process of 2′-FL

2′-FL used in this study was produced by Hongmo Biotech Co., Ltd. 2′-FL is produced through microbial fermentation using genetically modified production strains engineered for high-efficiency 2′-FL biosynthesis. During the fermentation process, D-glucose and D-lactose serve as the carbon source and substrate, respectively, to generate 2′-FL, which is released into the fermentation medium. The entire fermentation process is carried out under sterile conditions, with temperature and pH precisely controlled. At the end of the fermentation process, the bacterial biomass is inactivated by heat treatment followed by microfiltration to remove cells. The purification process includes ultrafiltration, ion exchange, decolorization, and concentration-crystallization steps. The final product obtained after fermentation, purification, and crystallization is purified 2′-FL in powdered form.

### 4.2 Acute oral toxicity test

The acute oral toxicity was based on the National Standard for Food Safety - Acute Oral Toxicity Test (GB 15193.3–2014). A total of 20 healthy SPF-grade ICR mice (10 males, 10 females, 18–22 g) were acclimatized for 5 days under controlled conditions (temperature 20 °C–26 °C, relative humidity 40%–70%, 12 h light/dark cycle) with free access to standard chow and water. Fasting for 4 h prior to the experiment with free access to water. The subjects were given oral intragastric administration once within 24  h, with an intragastric volume of 20 mL/kgbw, equivalent to an intragastric dose of 10.00 g/kgbw. Animals were then monitored over 14 days for any signs of acute toxicity, including body weight changes, failure to thrive, changes in food or water intake, or other clinical symptoms.

### 4.3 Bacterial reverse mutation (Ames test)

The bacterial reverse mutation test was performed according to The National Standard for Food Safety - Bacterial reverse mutation Test (GB 15193.4–2014). Five histidine-deficient strains of *Salmonella Typhimurium* (TA97a, TA98, TA100, TA102 and TA1535) were used to assess the mutagenic potential of 2′-FL. A mutagenicity test was conducted with the plate incorporation method at five different dose levels, both in the presence or absence of metabolic activation system (S9 mixture). For each dose level, 0.1 mL of bacterial suspension, 0.1 mL of test substance solution, and 0.5 mL of S9 mixture (or phosphate buffer for tests without S9) were added to 2.0 mL of molten top agar supplemented with histidine and biotin. After thorough mixing, the mixture was rapidly poured onto the surface of the minimal agar plates. Following incubation at 37 °C for 48 h, the number of revertant colonies per plate was counted. The first experiment showed negative results. For the confirmatory test, the experiment was repeated with a fivefold dose increment design. The doses tested were 5,000.0, 1,000.0, 200.0, 40.0, and 8.0 μg/plate. A vehicle control group, an untreated control group, and positive control groups (2-amino fluorene, Natriumazid, Dexon, 1,8-Dihydroxyanthraquinone, cytoxan) were included in the study. No reproducible or dose-related increases in revertant colony numbers (less than twofold increase) were observed with any strain following exposure to 2′-FL at any concentration. Three parallel doses were set for each dose.

### 4.4 Mammalian erythrocyte micronucleus test

Mammalian erythrocyte micronucleus test was conducted according to The National Standard for Food Safety - Mammalian Erythrocyte Micronucleus Test (GB 15193.5–2014). A total of 50 healthy SPF-grade ICR mice (25 males, 25 females, 25–35 g) were randomly divided into five groups, including a negative control group, three treatment group, and a positive control group. Mice were treated orally with 2′-FL twice at a 24-h interval. The experimental doses were at dose of 2.5, 5.0, 10.0 mg/kgbw. The negative control group received an equivalent volume of the vehicle, and the positive control group received a known clastogen (cyclophosphamide). All doses were administered at a constant volume of 20 mL/kg body weight. Femoral bone marrow was taken, diluted with calf serum, fixed with methanol, stained with Giemsa, and then examined by microscopy. The number of polychromatophilic erythrocytes (PCE) in 200 RBCS of each animal was counted and their proportion was calculated. The incidence of micronucleated polychromatic erythrocytes was determined by examining 2000 PCEs per animal under microscopy. A total of 2000 PCEs per animal were scored to calculate the micronucleus frequency (‰).

### 4.5 Mammalian spermatogonial chromosome aberration test

The mammalian spermatogonial chromosome aberration test was conducted according to The National Standard for Food Safety - Mammalian Spermatogonial Chromosome Aberration Test (GB 15193.8–2014). A total of 30 healthy SPF-grade ICR male mice (25–35 g) were randomized into five treatment groups (5 mice in each group except for 10 mice in the high-dose group). Subjects were administered orally by gavage once daily at 24-h intervals. The positive control group received an intraperitoneal injection of a known clastogen (cyclophosphamide). All administrations were performed at a constant volume of 20 mL/kg body weight. Epididymal tissues were collected to prepare a filtrate mear which was stained using Giemsa and evaluated via light microscope. For each animal, at least 1,000 spermatogonial cells were examined to determine the mitotic index. Chromosome structural aberrations were analyzed under a light microscope. The types and numbers of aberrations were recorded, including chromatid-type aberrations (breaks, exchanges, gaps) and chromosome-type aberrations (breaks, dicentrics, rings, fragments). The percentage of cells with aberrations and the number of aberrations per cell were calculated.

### 4.6 90-day subchronic toxicity test

The subchronic oral toxicity was based on The National Standard for Food Safety - 90-day Oral Toxicity Test (GB 15193.13–2015). Studies of subchronic toxicity were conducted using 100 healthy SPF-grade Sprague-Dawley (SD) rats (50 males, 50 females, 50–100 g). Rats were separated into three dose groups and one solvent control group (10 males and 10 females per group) and were orally administered samples for 90 consecutive days at three dose levels (2000, 4,000, or 8,000 mg/kgbw). All rats were monitored for clinical changes and mortality daily. Body weight and food intake were assessed weekly. At the same time, a hematology examination was performed. At the end of the treatment period, blood samples from the angular vein were collected to measure hematological parameters. Hematological parameters measured included red blood cell count (RBC), hemoglobin concentration (HGB), hematocrit (HCT), platelet count (PLT), white blood cell count (WBC) and differential (neutrophils, lymphocytes). For clinical biochemistry, blood samples were centrifuged to obtain plasma. The following parameters were measured using an automated biochemical analyzer: alanine aminotransferase (ALT), aspartate aminotransferase (AST), blood urea nitrogen (BUN), creatinine (CRE), fasting glucose (GLU), total protein (TP), albumin (ALB), total cholesterol (TC), and triglycerides (TG) After dissection, the liver, kidneys, spleen, and testes were collected and weighed, and Organ-to-body weight ratios were determined. In addition, the livers, kidneys, spleens, stomachs, duodenums, testes, and ovaries of the animals in the control and the high-dose group were fixed and subjected to histopathological assessment.

### 4.7 Teratogenicity test

The teratology was based on The National Standard for Food Safety - Teratogenicity Test (GB 15193.14–2015). Studies of teratogenicity test were conducted using 100 healthy Sprague-Dawley (SD) rats (70 males, 140 females, 220–260 g). The subjects were divided into four groups, and the dose of 8.00 g/kgbw, which was not observed adverse effect level (NOAEL) in the 90-day subchronic toxicity test, was set as the high dose, the medium dose was set as 4.00 g/kgbw, and the low dose was set as 2.00 g/kgbw. Pregnant rats were weighed, randomly assigned to each group, and numbered, weighed at the sixth, ninth, 12th, 15th and 20th days of pregnancy, and given once a day by oral gavage from the sixth to the 15th day of pregnancy. The rats were killed on the 20th day of pregnancy, and the uterus was removed by laparotomy and weighed. Gross anatomical examination was performed to check the corpus luteum, absorption fetus, early stillbirth, late stillbirth and number of live births. The sex, weight and body length of the fetuses were recorded, and the appearance of the fetuses was checked for abnormalities.

### 4.8 Statistical analyses

Data statistics were analyzed using SPSS 19.0 software. For continuous variables (measurement data), data are presented as mean ± standard deviation (SD). For comparisons between multiple experimental groups and the control group, Dunnett’s t-test was employed. For categorical variables (count data), the chi-square (χ^2^) test was used.

## Data Availability

The original contributions presented in the study are included in the article/Supplementary Material, further inquiries can be directed to the corresponding author.
