# Supplementary material for: A comprehensive safety evaluation of approved 2′-fucosyllactose for human nutritional applications in China
Source: Front Toxicol. 2026 Jun 23;8:1836564. doi: 10.3389/ftox.2026.1836564 (PMC13336670; doi:10.3389/ftox.2026.1836564)
Supplement: Supplementary file 1 [file DataSheet1.doc]

Supplementary Material


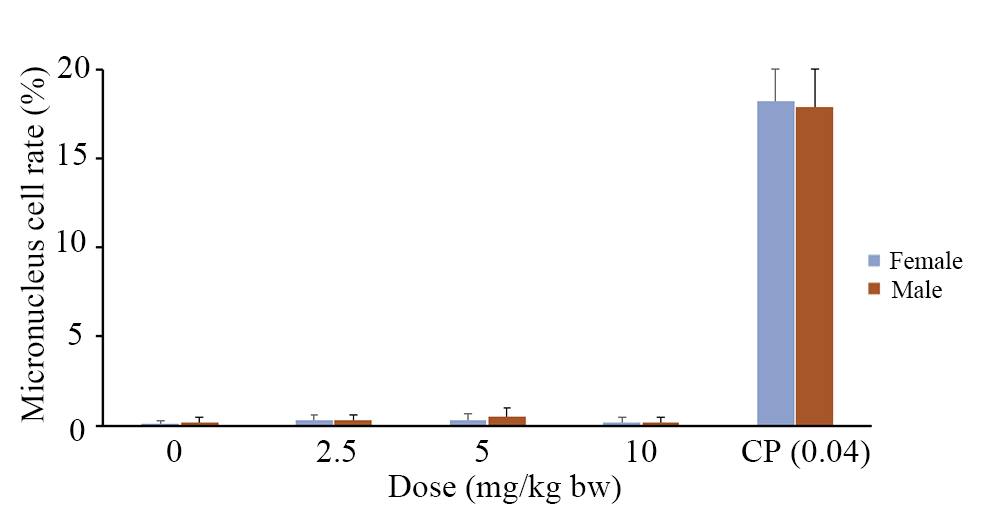


**Supplementary Figure 1**. Micronucleus test in mouse erythrocytes with 2’-FL. CP represents cyclophosphamide, at a concentration of 0.04 mg/kg bw. Compared with the negative control group, the micronucleus cell rate of both female and male mice in the positive control group (cyclophosphamide) showed a statistically significant difference (*P*<0.01). In contrast, no significant differences were observed in the micronucleus cell rate of either female or male mice in any dose groups of the test substance when compared with the negative control group (*P*>0.05).


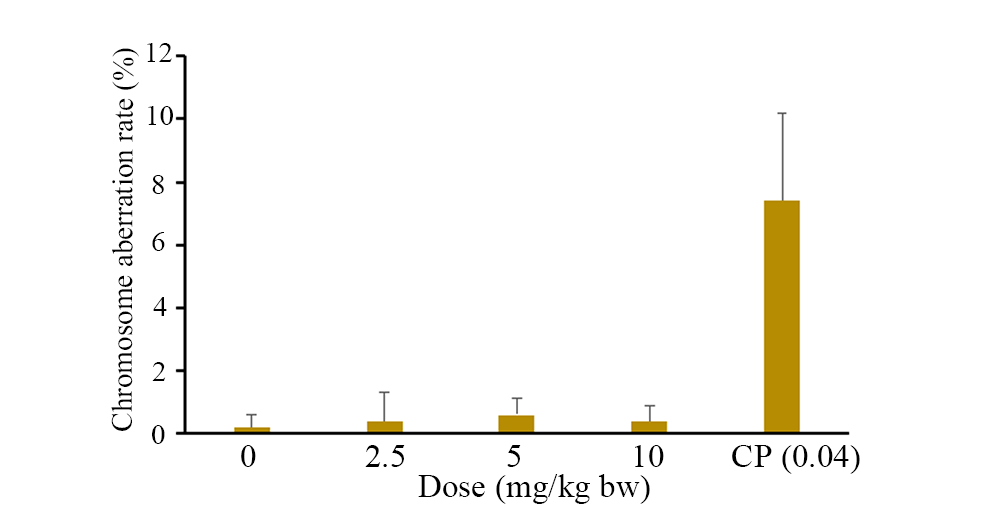


**Supplementary Figure 2**. The chromosomal aberration test in mammalian spermatogonial cells *in vitro* with 2’-FL. CP represents cyclophosphamide, at a concentration of 0.04 mg/kg bw. Compared with the negative control group, the chromosomal aberration rate in spermatogonia of mice in the positive control group (cyclophosphamide) showed a statistically significant difference (*P*<0.01). In contrast, no significant difference was observed in the chromosomal aberration rate in spermatogonia of mice across all dose groups of the test substance when compared to the negative control group (*P*>0.05).


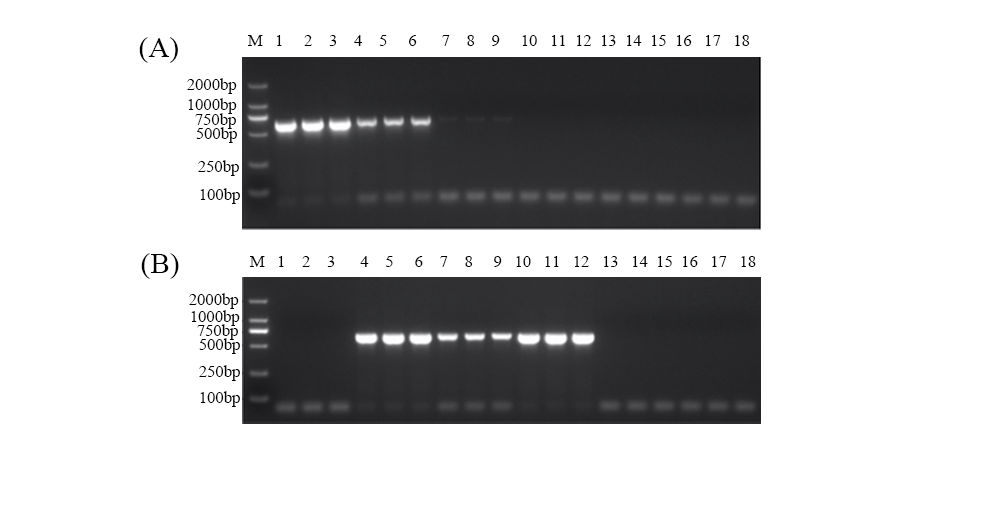


**Supplementary Figure 3.** Detection of exogenous gene residues in the final 2’-FL product.

For the detection of exogenous gene residues in the final 2’-FL product, the futC gene, which encodes the core enzyme α-1,2-fucosyltransferase, was selected as the representative PCR target.

1. Sensitivity analysis. Lane numbers 1-18 are arranged as follows: lanes 1-3, 100.00 ng production strain/g sample; lanes 4-6, 10.00 ng production strain/g sample; lanes 7-9, 1.00 ng production strain/g sample; lanes 10-12, 0.10 ng production strain/g sample; lanes 13-15, 0.01 ng production strain/g sample; lanes 16-18, blank controls. The detection limit of the method was determined to be 10 ng production strain/g sample.

(B) Exogenous gene residue detection in the final 2’-FL product. Lane numbers 1-18 are arranged as follows: lanes 1-3, 2’-FL test samples; lanes 4-6, positive controls (1.00 ng/μL, production stain DNA); lanes 7-9, spike-and-recovery samples (1 ng DNA/g sample); lanes 10-12, quality controls (1.00 ng/μL, sample DNA spiked with production strain DNA); lanes 13-15, negative controls (1.00 ng/μL, non-recombinant *E. coli* BL21(DE3) DNA); lanes 16-18, blank controls (ddH_2_O). The expected amplicon size is 643 bp. No exogenous gene residues were detected in the final 2’-FL product (lanes 1-3).


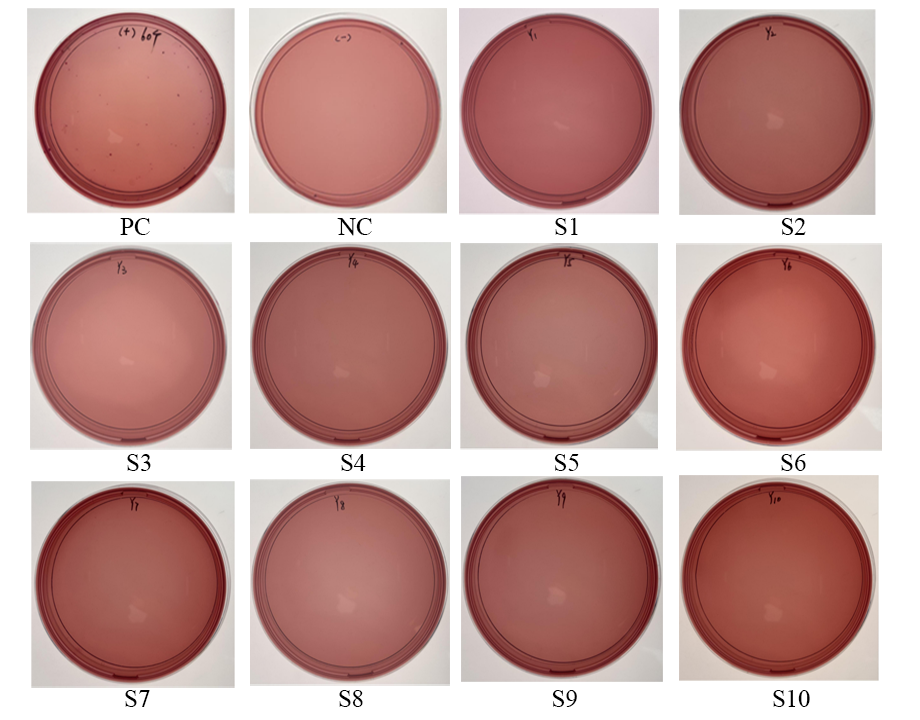


**Supplementary Figure 4.** Detection of viable bacteria in 2’-FL products. Samples were plated on VRBA and incubated at 37℃ for 24 h. Negative control (NC): sterile PBS. Positive control (PC): production strain *E. coli* BL21(DE3). Ten parallel samples (S1-S10) each showed no bacterial colonies, identical to NC. No viable bacteria were detected in any test sample.

**Supplementary Table 1**. Organ weights, organ/body weight ratios of rats sacrificed on the 90^th^ day in the subchronic test (mean±SD, n=10)

| **Organ** | **Organ weight (g)** | | | | **Organ/body weight ratios (g/100g)** | | | |
| --- | --- | --- | --- | --- | --- | --- | --- | --- |
|  | **Control** | **Low** | **Middle** | **High** | **Control** | **Low** | **Middle** | **High** |
| Female | | | | | | | | |
| Brain | 1.650±0.099 | 1.614±0.136 | 1.620±0.139 | 1.595±0.080 | 0.450±0.040 | 0.460±0.060 | 0.450±0.040 | 0.420±0.040 |
| Heart | 1.088±0.162 | 1.152±0.073 | 1.163±0.122 | 1.148±0.124 | 0.298±0.045 | 0.327±0.030 | 0.324±0.043 | 0.303±0.018 |
| Liver | 9.292±1.276 | 9.183±0.852 | 9.584±0.759 | 9.624±0.756 | 2.540±0.320 | 2.600±0.150 | 2.660±0.200 | 2.540±0.130 |
| Kidney | 2.147±0.208 | 2.154±0.156 | 2.149±0.159 | 2.210±0.221 | 0.590±0.090 | 0.610±0.050 | 0.600±0.060 | 0.580±0.050 |
| Spleen | 0.730±0.102 | 0.693±0.106 | 0.709±0.080 | 0.670±0.117 | 0.199±0.020 | 0.197±0.033 | 0.197±0.024 | 0.177±0.026 |
| uterus | 0.724±0.263 | 0.774±0.251 | 0.716±0.230 | 0.700±0.201 | 0.200±0.080 | 0.220±0.090 | 0.200±0.060 | 0.190±0.060 |
| Ovarian | 0.208±0.051 | 0.217±0.045 | 0.206±0.051 | 0.221±0.042 | 0.057±0.013 | 0.062±0.014 | 0.057±0.013 | 0.058±0.009 |
| Thymus | 0.460±0.168 | 0.523±0.117 | 0.494±0.118 | 0.535±0.091 | 0.127±0.048 | 0.147±0.03 | 0.137±0.032 | 0.143±0.031 |
| Adrenal gland | 0.093±0.025 | 0.097±0.027 | 0.105±0.026 | 0.102±0.025 | 0.026±0.009 | 0.027±0.007 | 0.029±0.007 | 0.027±0.006 |
| Male | | | | | | | | |
| Brain | 1.650±0.291 | 1.724±0.119 | 1.652±0.122 | 1.682±0.134 | 0.270±0.060 | 0.280±0.030 | 0.270±0.030 | 0.260±0.020 |
| Heart | 1.869±0.248 | 1.729±0.204 | 1.857±0.243 | 1.855±0.289 | 0.303±0.048 | 0.285±0.034 | 0.302±0.019 | 0.287±0.027 |
| Liver | 17.589±3.093 | 16.729±2.204 | 15.762 4.169 | 17.176±3.366 | 2.850±0.610 | 2.760±0.350 | 2.580±0.620 | 2.640±0.280 |
| Kidney | 3.901±0.297 | 3.764±0.494 | 3.757±0.550 | 3.699±0.363 | 0.630±0.090 | 0.620±0.070 | 0.610±0.070 | 0.580±0.100 |
| Spleen | 1.023±0.129 | 0.955±0.114 | 1.037±0.212 | 1.065±0.141 | 0.165±0.020 | 0.157±0.019 | 0.167±0.016 | 0.165±0.015 |
| testicle | 3.736±0.226 | 3.173±0.566 | 3.709±0.312 | 3.746±0.305 | 0.610±0.090 | 0.530±0.110 | 0.610±0.060 | 0.590±0.070 |
| epididymis | 1.531±0.194 | 1.357±0.351 | 1.336±0.459 | 1.591±0.173 | 0.250±0.050 | 0.220±0.060 | 0.220±0.070 | 0.250±0.030 |
| Thymus | 0.631±0.195 | 0.661±0.171 | 0.620±0.116 | 0.639±0.141 | 0.101±0.029 | 0.110±0.031 | 0.102±0.019 | 0.100±0.024 |
| Adrenal gland | 0.098±0.030 | 0.076±0.022 | 0.084±0.034 | 0.098±0.030 | 0.016±0.004 | 0.012±0.004 | 0.014±0.007 | 0.015±0.004 |
